# The value of plasma insulin-like growth factor 1 and interleukin-18 in the diagnosis of bronchopulmonary dysplasia in premature infants

**DOI:** 10.3389/fped.2022.1013537

**Published:** 2022-10-11

**Authors:** Lie Huang, Ning Guo, Meile Cheng, Jianhui Wang, Feifan Chen, Yuan Shi

**Affiliations:** ^1^ Department of Neonatology, Children’s Hospital of Chongqing Medical University, National Clinical Research Center for Child Health and Disorders, Ministry of Education Key Laboratory of Child Development and Disorders, Chongqing Key Laboratory of Pediatrics, Chongqing, China; ^2^ Department of Neonatology, The First People’s Hospital of Yinchuan, Ningxia Medical University, Yinchuan, China

**Keywords:** bronchopulmonary dysplasia, very low birth weight (VLBW), IGF-1 (insulin-like growth factor 1), IL-18, inflammation

## Abstract

**Objective:**

To explore the diagnostic value of IGF-1 and IL-18 in premature infants with BPD.

**Methods:**

Through a prospective observational study, the serum samples of infants in the BPD group and the non-BPD group were collected at different targeted time points, and the serum IGF-1 and IL-18 concentrations were dynamically monitored by ELISA. The Student *t*-test and one-way analysis of variance were adopted to analyze data, and the receiver operating characteristic (ROC) curve was used to test the diagnostic value.

**Result:**

A total of 90 VLBW premature infants admitted to NICU between January 2020 and 2021 were finally included. Compared with the non-BPD group, infants diagnosed with BPD had a significantly lower serum concentration of IGF-1 (*P* < 0.05) but a higher level of IL-18 (*P* < 0.05) on days 1, 7, 14, and 28 after birth. With the ROC curve analysis, the serum concentration IGF-1 on day 14 and IL-18 on day 28 reported high sensitivity and specificity to predict the risk of BPD (IGF-1: sensitivity: 89.29%, specificity: 77.78%, AUC: 0.8710; IL-18: sensitivity: 53.57%, specificity: 83.33%, AUC: 0.7887). And more substantial predictive power was found in combined analysis of IGF-1 and serum IL-18 on day 14: the sensitivity was 91.07% and the specificity was 83.33%, with the AUC of 0.9142.

**Conclusion:**

IGF-1 and IL-18 might be closely involved in the occurrence and development of BPD. The serum concentration of IGF-1 combined with IL-18 could be potentially sensitive markers for the early diagnosis and severity of BPD.

## Introduction

Bronchopulmonary dysplasia (BPD), a chronic neonatal lung disease, is one of the most common and severe sequelae of premature births (1–5). Up to 15%–25% of infants born at <32 weeks of gestational age and 60% of those born at <28 weeks will develop BPD, with potential adverse events that can persist into their adulthood and lead to lifelong respiratory and neurodevelopmental consequences (6, 7).

However, the pathogenesis of BPD remains unclear and specific treatments are still lacking, so preventive measures and early diagnosis are indeed crucial. The pathogenesis of BPD is multifactorial (8, 9), involving immature lung tissue, excessive inflammatory injury, and abnormal repair processes, especially the abnormal repair after injury may significantly impair the development of airways, lung parenchyma, pulmonary interstitial, pulmonary vessels, and lymphatic system (10). Many previous studies have reported that growth factors and cytokines involved in the early immune response can initiate the immune cascade signaling pathways of the inflammatory response, participate in the occurrence and progression of BPD, and ultimately may affect the pulmonary structure of infants (11).

Insulin-like growth factor 1 (IGF-1) is a key factor in embryonic and postnatal growth and development, including stimulating cell proliferation and differentiation, inhibiting apoptosis, and regulating gene transcription (12), which is also found to play an important role in all stages of lung differentiation and maturation. IGF-1 can help repair and remodel airways by promoting the proliferation and differentiation of airway epithelial cells and fibroblasts (13). IGF-1 knockout mice exhibit impaired lung development and maturation, and neonatal rats exposed to hyperoxia are detected with decreased IGF-1 expression levels in the lungs and inhibited alveolarization processes (14). Besides IGF-1, Interleukin-18 (IL-18), a pro-inflammatory mediator and a member of the IL-1 cytokine family, maybe another potential predictor of BPD. It has been proved to be critical in many pulmonary diseases such as acute respiratory distress syndrome (ARDS) and chronic obstructive pulmonary disease (COPD) in adults (15). IL-18 can participate in the progression of lung inflammation by promoting TH1-type helper T-cell responses and promote fibroblast proliferation and collagen deposition in the pulmonary fibrosis process (16).

All the above findings suggest that IGF-1 and IL-18 may participate in the occurrence and development of BPD. This study aimed to explore the relationship between IGF-1 and IL-18 and BPD by detecting the serum concentration, hoping to provide a new reference for possible biomarkers for BPD prediction in premature infants.

## Materials and methods

### Patient enrollment

The study population consisted of neonates admitted to the NICU of The First People's Hospital of Yinchuan between January 2020 and 2021, with a gestational age <32 weeks, and hospitalized for more than 28 days. Infants with major congenital malformations, the age more than 24 h upon admission to the NICU, severe infection, shock, inherited metabolic diseases and who withdrew during the study period were excluded. All the neonates were divided into two groups: the BPD group and the non-BPD group. Infants were diagnosed with BPD according to the criteria from the workshop of the National Institutes of Child Health and Human Development (NICHD) (17) and were classified as follows: mild: breathing room air, moderate: a fraction of inspired oxygen FiO_2_<0.3, severe: FiO_2_ ≥0.3 and/or positive pressure ventilation or mechanical ventilation.

This study was approved by the ethics committee of The First People's Hospital of Yinchuan (Number: 2020110). Informed consent was obtained from all the parents of the included infants.

### Collection of clinical data

The following data were collected from hospital records: (1) general condition of the infants: gender, gestational age (according to the first day of the last menstrual period) at birth, birth weight, Apgar Score 1 min, Apgar Score 5 min, (2) maternal conditions: Chorioamnionitis, Premature rupture of membranes, Antenatal steroids, Gestational hypertension, Gestational diabetes, (3) birth injuries, conditions, and comorbidities present in the infants: patent ductus arteriosus, late-onset neonatal sepsis, necrotizing enterocolitis and so on.

### Sample collection

Detection of serum IGF-1 and IL-18 levels: 1–1.5 ml of neonatal serum samples were collected each time from all the infants on days 1, 7, 14, and 28 after birth. The serum samples were transferred to Eppendorf tubes and stored at −20° C until assayed. Concentrations of IGF-1 and IL-18 were detected using commercial enzyme-linked immunoassay (ELISA) according to the manual instructions strictly.

### Statistical analysis

Refer to the relevant literature (18), the sample size was calculated based on the mean serum value of IL-18 and IGF-1 (213, 37), and the estimation was conduct with G*Power 3.1.7, with 75% power and a 2-sided *α* of 0.05 considering clinically significant. The finally required number of samples was about 88.

Statistical analysis was performed using SPSS24.0 statistical software. All quantitative variables were assessed for the normality test and described as the means ± standard deviations (-x ± s) if conformed to a normal distribution. All qualitative variables were described as frequencies or percentages. For comparisons between two groups, the Student t-test was adopted for normally distributed variables, and the Mann-Whitney U test was used when the normality test failed. In the multiple-group comparisons, repeated measurement one-way analysis of variance (ANOVA) was adopted for normally distributed variables, and the non-parametric Kruskal-Wallis test was used when the normality test failed. Diagnostic efficacy for predicting the risk of BPD was evaluated by the receiver operating characteristic (ROC) analysis (AUROC of 0.5 indicates no capacity for differentiation and 1.0 indicates perfect differentiation). *P* < 0.05 was considered statistically significant in all tests.

## Results

### Patient characteristics

**Figure 1:**
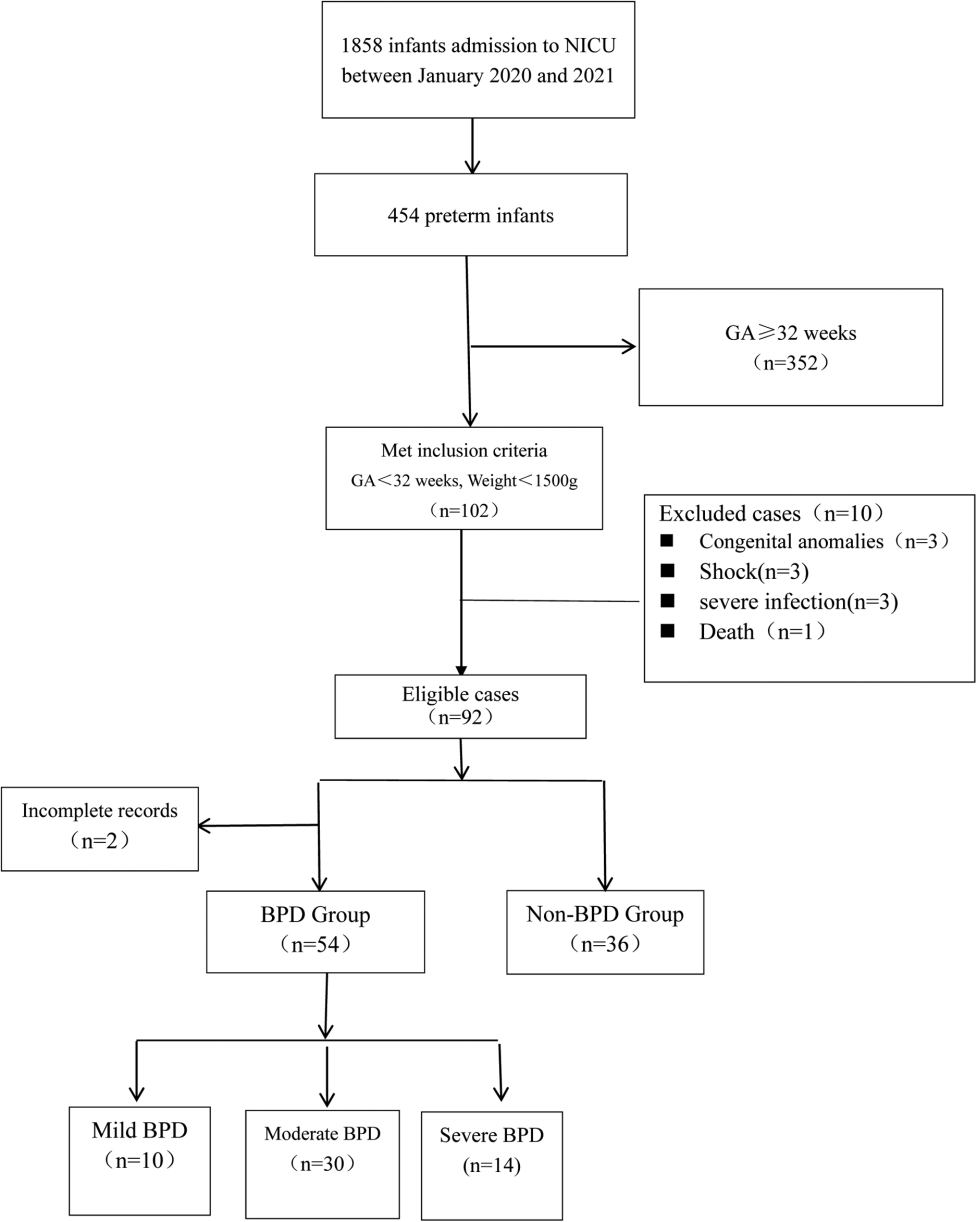
Flow chart for patient selection.

In the recruitment, the information of 102 patients was obtained from The First People's Hospital of Yinchuan and 90 premature infants were finally enrolled in the study (Figure 1**)**. In the BPD group, 54 newborns were diagnosed with BPD, including 33 males (61.1%) and 21 females (38.9%), with a mean birth weight of 1332 ± 321.5 grams and a mean gestational age of 30.18 ± 1.36 weeks, of which 10 infants were categorized as mild BPD and 30 infants were categorized as moderate BPD and the other 14 infants were categorized as severe BPD. 36 non-BPD premature infants were included in the control group, with a mean birth weight of 1396 ± 237.8 grams and a mean gestational age of 30.28 ± 1.34 weeks. No significant difference in demographic data at birth was reported between the two groups (*P* > 0.05). The characteristics of the population are presented in Table 1.

**Table 1:** Baseline characteristics of included 90 infants.

Variable	BPD (*n* = 54)	Non-BPD (*n* = 36)	*P*
Infant characteristics			
Birth weight (gram), mean (SD)	1332 ± 321.5	1396 ± 237.8	0.31
Gestational age (week), mean (SD)	30.18 ± 1.36	30.28 ± 1.34	0.73
Gender (male), *n* (%)	33 (61.1)	18 (50)	0.3
Apgar 1min, median (IQR)	4 (0-8)	5 (0-9)	0.07
Apgar 5min, median (IQR)	6 (3-9)	7 (3-10)	0.065
Patent ductus arteriosus, *n* (%)	27 (50)	9 (25)	0.018
Late-onset neonatal sepsis, *n* (%)	14 (25.9)	8 (22.2)	0.69
Necrotizing enterocolitis, *n* (%)	3 (5.5)	1 (2.7)	0.65
Maternal characteristics			
Chorioamnionitis, *n* (%)	12 (22.2)	4 (11.1)	0.18
Premature rupture of membranes, *n* (%)	15 (27.7)	6 (16.7)	0.22
Antenatal steroids, *n* (%)	27 (50)	28 (77.8)	0.008
Gestational hypertension, *n* (%)	6 (11.1)	2 (5.5)	0.47
Gestational diabetes, *n* (%)	4 (7.4)	3 (8.3)	1

### Serum IGF-1 concentrations in the BPD group and the non-BPD group

**Figure 2:**
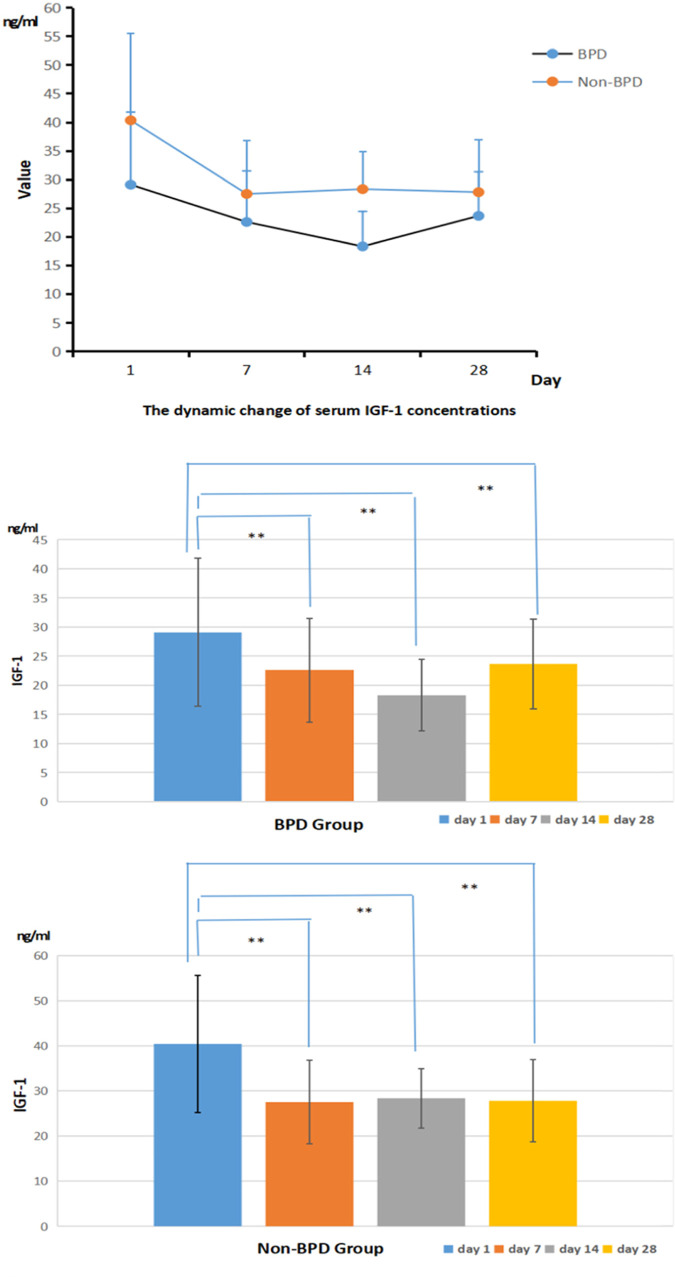
The dynamic serum IGF-1 concentrations in the BPD and the non-BPD group (ng/ml).

The concentrations of IGF-1 in serum samples in both the BPD and the non-BPD groups reached the peak value on day 1 and gradually decreased after day 1. The significantly lower level of IGF-1 in infants in the BPD group was always observed than that in the non-group on days 1, 7, 14, and 28 (*P* < 0.05). In the BPD group, the highest serum level of IGF-1 was on day 1 and the lowest was on day 14, and the value on days 7 and 14 were significantly lower than that on day 1 (*P* < 0.05). In the non-BPD group, the serum IGF-1 level reached the highest on day 1 and the lowest on day 7, and the value on days 7, 14, and 28 were significantly lower than that on day 1 (*P* < 0.05). All the details were shown in Figures 2.

### Serum IGF-1 concentrations in infants with different severity of BPD

**Figure 3:**
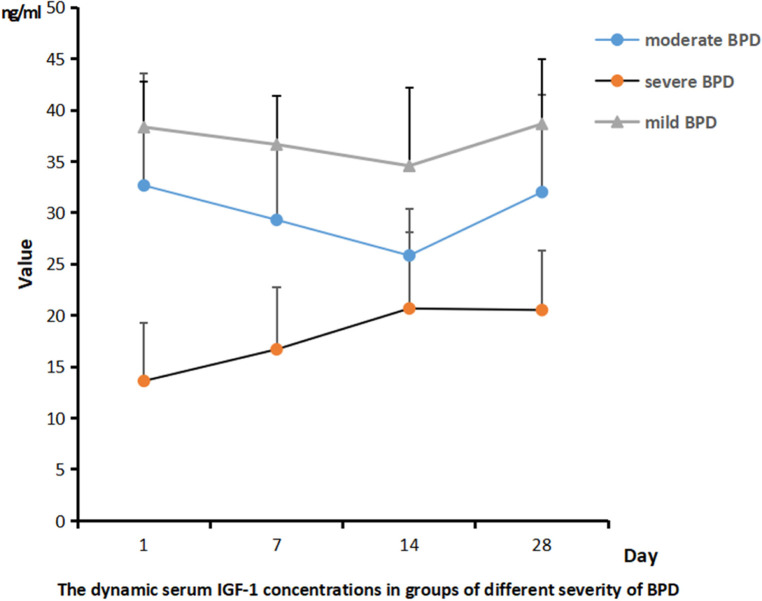
The dynamic serum IGF-1 concentrations in groups of different severity of BPD.

As shown in Figures 3, the concentrations of IGF-1 in serum samples from infants with different severity of BPD were always different on days 1, 7, 14, and 28, and infants diagnosed with severe BPD were significantly associated with a lower level of IGF-1 than those with mild BPD (*P* < 0.05).

### Serum il-18 concentrations in the BPD group and the non-BPD group

**Figure 4:**
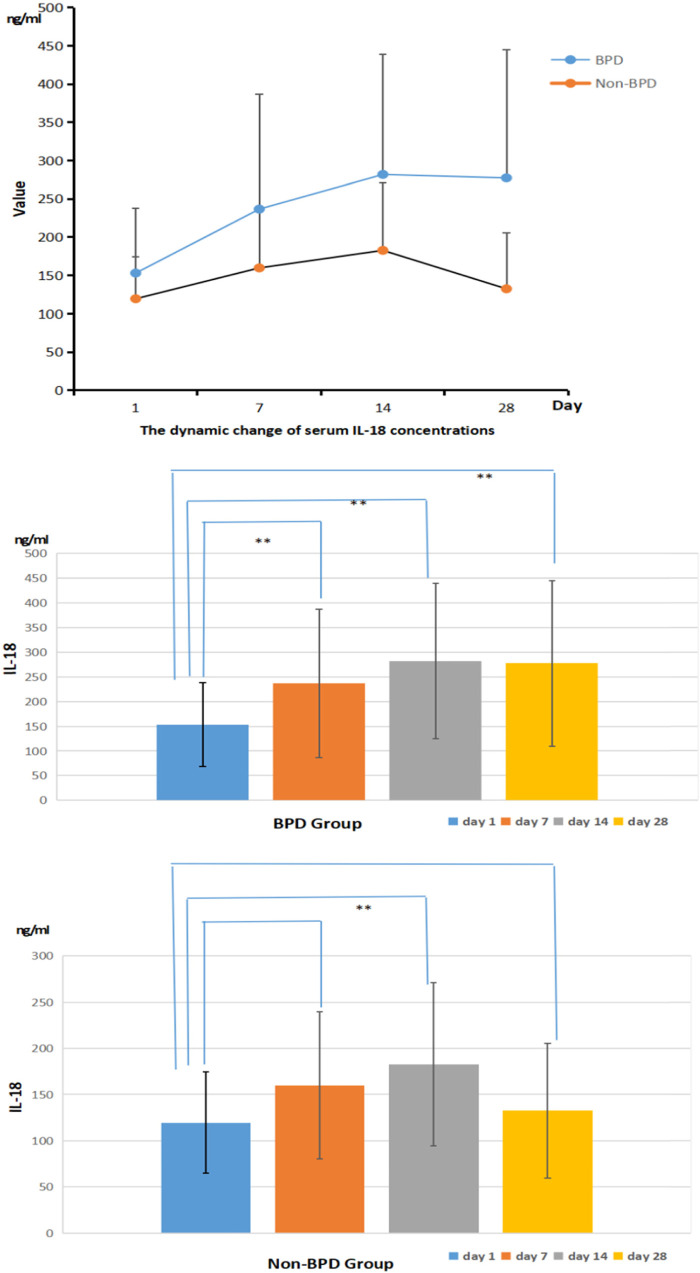
The dynamic serum IL-18 concentrations in the BPD and non-BPD group (ng/ml).

The concentration of IL-18 in the serum samples from both the BPD group and the non-BPD group shared the same trend: the lowest value was on day 1, increased and reached the peak value on day 14, and then decreased gradually. Significantly higher concentration of serum IL-18 in the BPD group was always observed than that in the non-BPD group on days 1, 7, 14, and 28 (*P* < 0.05). In the BPD group, the serum IL-18 concentration on day 1 was significantly lower than that on days 7, 14, and 28 (*P* < 0.05). In the non-BPD group, the serum IL-18 concentration on day 14 was significantly higher than that on day 1 (*P* < 0.05). Details were shown in Figures 4.

### Serum il-18 concentrations in infants with different severity of BPD

**Figure 5:**
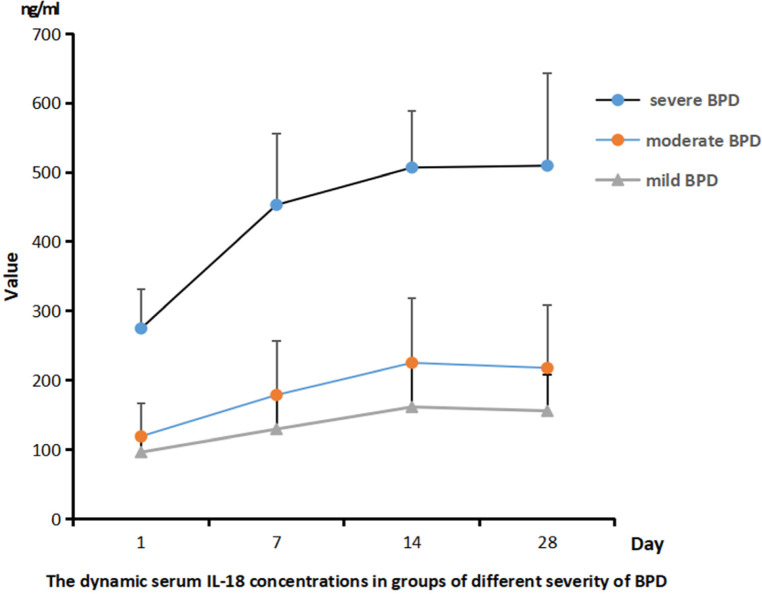
The dynamic serum IL-18 concentrations in groups with different severity of BPD (ng/ml).

As shown in Figures 5, the serum level of IL-18 in infants with different severity of BPD were always different on days 1, 7, 14, and 28, and infants with severe BPD were significantly associated with a higher serum level of IL-18 than those with mild BPD (*P* < 0.05).

### The diagnostic value of serum IGF-1 for BPD

**Figure 6:**
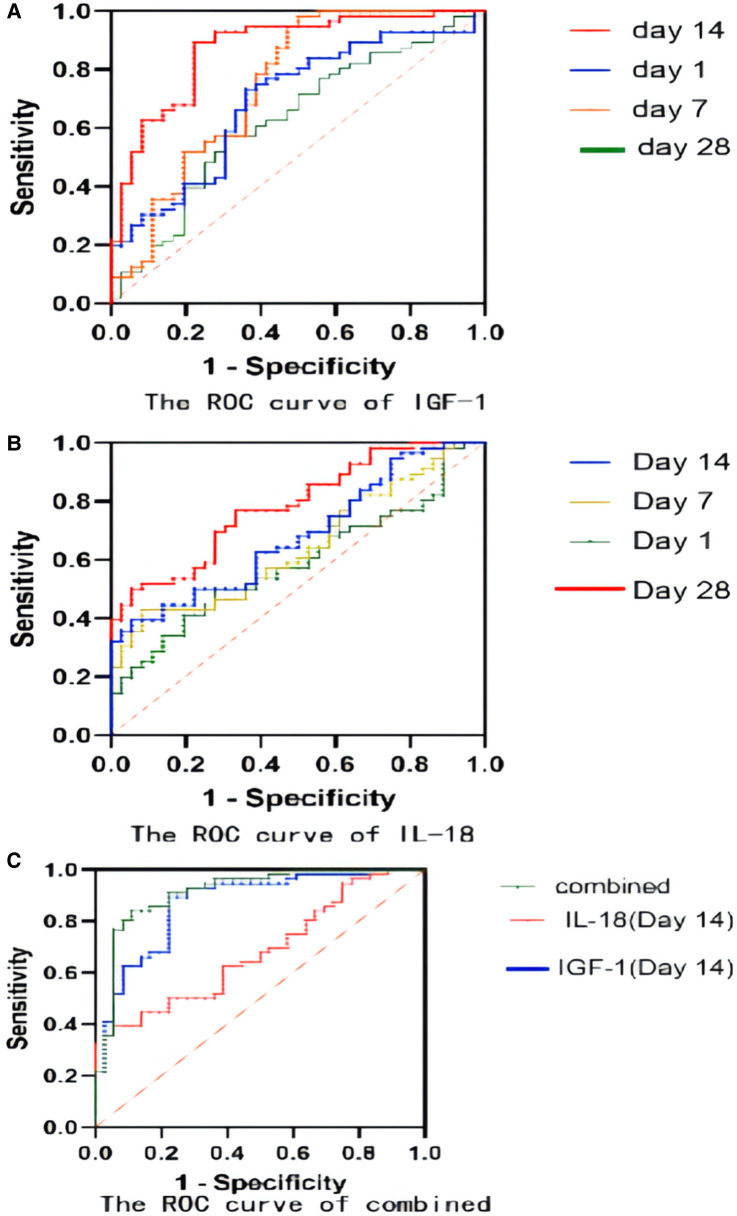
(**A**) The ROC curve of IGF-1 for BPD prediction; (**B**) The ROC curve of IL-18 for BPD prediction; (**C**) The ROC curve of IGF-1 combined with IL-18 for BPD prediction.

With the ROC curve analysis, the diagnostic value of serum IGF-1 for BPD at different time points were shown in Table 2 and Figure 6A. Serum IGF-1 concentrations on day 14 had a good predictive power with a cut-off value of 23.4 ng/ml (AUC: 0.871, sensitivity: 89.29%, specificity: 77.78%, *P* < 0.0001).

**Table 2:** Diagnostic value of IGF-1 for predicting BPD at different time points.

Index	AUC	Sensitivity (%)	Specificity (%)	Cut-off value	*P*
Day 1	0.7475	87.5	74.75	36.43	<0.0001
Day 7	0.6915	73.21	63.89	24.12	0.002
Day 14	0.871	89.29	77.78	23.4	<0.0001
Day 28	0.626	57.14	62.60	24.33	<0.0001

### The diagnostic value of serum il-18 for BPD

Table 3 and Figure 6B demonstrated the results of serum IL-18 for BPD prediction at different time points. Serum IL-18 concentrations on day 28 had a good predictive power with a cut-off value of 248 ng/ml (AUC: 0.7837, sensitivity: 53.57%, specificity: 83.33%, *P* < 0.05).

**Table 3:** Diagnostic value of IL-18 for predicting BPD at different time points.

Index	AUC	Sensitivity (%)	Specificity (%)	Cut-off value	*P*
Day 1	0.5903	50	72.22	135.1	0.1454
Day 7	0.6443	42.86	91.67	243.3	0.0199
Day 14	0.6815	51.79	77.78	314.9	0.0034
Day 28	0.7837	53.57	83.33	248	0.04

### The diagnostic value of IGF-1 combined with il-18 for predicting BPD

For a better prediction of BPD, a combined analysis of serum IGF-1 concentration and serum IL-18 concentration on day 14 was conducted in Table 4 and Figure 6C: the sensitivity was 91.07% and the specificity was 83.33%, with the AUC of 0.9142, which demonstrated more substantial predictive power than that in a single variable included analysis.

**Table 4:** The diagnostic value of IGF-1 combined with IL-18 for predicting BPD.

Index	AUC	Sensitivity (%)	Specificity (%)	*P*
IGF-1 (day 14)	0.871	89.29	77.78	<0.0001
IL-18 (day 14)	0.6815	51.79	77.78	0.0034
Combined	0.9142	91.07	83.33	<0.0001

## Discussion

BPD is a chronic lung disease in premature infants, which has been increasingly recognized to result from pathological repair responses to prenatal and postnatal injuries during lung maturation. Various patterns of prenatal stress combined with different postnatal injuries may significantly impair the development of the airways, lung parenchyma, pulmonary interstitial, lymphatic system, and pulmonary vessels. In infants with severe BPD, cessation of alveolarization may persist into late childhood and lead to permanent injuries. Currently, lung inflammation has been considered central to many theories of etiology and pathogenesis of BPD. Chorioamnionitis, oxygen toxicity, mechanical ventilation, and postnatal infections can induce inflammatory responses in the immature airways and lung tissue (19). IGF-1 has been confirmed to involve in the growth and injury repair processes of many organs, including the lung (20). A previous study reported the positive expression of IGF-1 and its receptor in the processes of injury and repair of alveolar type II cells, and thus hypothesized that IGF-1 might play an important role in the regulation of proliferation and differentiation of alveolar epithelial cells (21). IGF-1 is a downstream factor of the vascular endothelial growth factor (VEGF) signaling pathway, which can participate in the occurrence and progression of BPD by affecting alveolar microvascular formation (22). Some evidence put forward that IGF-1 might have the potential to modulate neonatal immune responses, and the expression of IGF-1 and its receptors could promote the development and maturation of the immune system (23). IGF-1 may also influence immune system homeostasis by promoting lymphangiogenesis and cell proliferation and differentiation, and participate in inflammation by stimulating inflammatory cytokines and chemokines as a pro-inflammatory factor.

In this study, the serum IGF-1 concentration was reported highest on the first day after birth and gradually decreased as time went by. Infants diagnosed with BPD were significantly associated with a lower serum IGF-1 level than those in the non-BPD group. The relationship between the severity of BPD and IGF-1 was also preliminarily analyzed and the result showed that infants with severe BPD usually had a lower expression IGF-1 level than those with mild BPD. The ROC analysis reported the highest sensitivity and specificity to predict the risk of BPD using the serum IGF-1 level on day 14. IL-18, a member of the IL-1 cytokine family, is a key pro-inflammatory mediator with particular importance in pulmonary infections and inflammation. IL-18 has been proposed as a novel biomarker for human ARDS, and its plasma level has been proved to correlate with ARDS severity and mortality. The organism can activate the NLRP3 inflammasome in response to infection, tissue damage, and oxidative stress, and then this activated complex transforms the cystatin-1 precursor into an active 20 kDa fragment that enables to promote of the IL-18 precursor into mature IL-18 and facilitate its release, which is considered significant in the initiation of inflammation (24). A growing number of studies have revealed that activation of the nucleotide-binding domain and leucine-rich repeat protein 3 (NLRP3) inflammasome is related to the pathogenesis of acute lung injury (25, 26). The activated NLRP3 inflammasome functions as a supramolecular platform for the caspase-1–dependent maturation and secretion of the proinflammatory cytokines IL-1β and IL-18 in macrophages (27, 28). Liao's research showed that NLRP3 activation is one of the primary causes of BPD (29). As our results shown, the concentration of IL-18 in the serum samples from both the BPD group and the non-BPD group shared the same trend: the lowest was on day 1, increased and reached the peak value on day 14, and then decreased gradually. The serum IL-18 concentration of the BPD group was significantly higher than that of the non-BPD group, and it also seemed to positively correlate with the clinical severity of BPD. Through the ROC analysis, the serum concentration of IGF-1 on day 14 and Il-18 on day 28 showed high sensitivity and specificity when predicting the risk of BPD respectively, which suggested their potential value in the diagnosis of BPD. With the combined analysis of IGF-1 on day 14 and IL-18 on day 14, the result reported the highest sensitivity (91.07%) and specificity (83.33%), also with the highest AUC (0.9142), which was superior to the prediction by IGF-1 and IL-18 alone. Compared with previous studies (30), it reported higher specificity and sensitivity. Consequently, the serum level of IGF-1 combined with the IL-18 may be more effective when establishing a predictive model of BPD. It is very convenient to collect sample from children clinically, and the detection of IL-18 and IGF-1 is also easy to conduct, which is feasible in clinical application.

However, this study has some limitations. The number of included patients was relatively small. Moreover, it was a single-center study, lacking multicenter clinical data support. Although it reported high sensitivity and specificity for predicting BPD, there is still a need for multicenter studies with larger sample sizes to improve the statistical power.

In conclusion, both IGF-1 and IL-18 might be closely involved in the occurrence and development of BPD. The decreased serum IGF-1 level and increased IL-18 level in preterm infants might be significantly associated with the severity of BPD. The serum concentration of IGF-1 combined with IL-18 could be potentially sensitive biomarkers for BPD prediction, but its diagnostic value still needs to be further verified in larger sample size research.

## Data Availability

The raw data supporting the conclusions of this article will be made available by the authors, without undue reservation.
